# Quantitative DSA Analysis of MCA Aneurysms Using SymDIRECT Pixel Clustering: A Novel Framework for Objective Post-Treatment Evaluation

**DOI:** 10.3390/diagnostics15162036

**Published:** 2025-08-14

**Authors:** Ante Rotim, Marina Raguž, Nikica Fulir, Darko Orešković, Vladimir Kalousek, Petar Marčinković, Krešimir Rotim, Bruno Splavski, Silva Butković Soldo, Tomislav Sajko

**Affiliations:** 1 Department of Neurosurgery, Sestre Milosrdnice University Hospital Center, 10 000 Zagreb, Croatia; ante.rotim92@gmail.com (A.R.);; 2 Faculty of Medicine, Josip Juraj Strossmayer University of Osijek, 31 000 Osijek, Croatia; 3 Department of Neurosurgery, Dubrava University Hospital, 10 000 Zagreb, Croatiapetar.marcinkovic11@gmail.com (P.M.); 4 School of Medicine, Catholic University of Croatia, 10 000 Zagreb, Croatia; 5 Department of Radiology, Sestre Milosrdnice University Hospital Center, 10 000 Zagreb, Croatia; 6 Special Hospital Neurospine, 10 000 Zagreb, Croatia; 7 University of Applied Health Sciences, 10 000 Zagreb, Croatia; 8 Department of Neurosurgery, Dubrovnik General Hospital, 20 000 Dubrovnik, Croatia; 9 Department of Neurology, University Hospital Center Osijek, 31 000 Osijek, Croatia

**Keywords:** digital subtraction angiography, SymDIRECT, image clustering, MCA aneurysm, vascular remodeling, postoperative imaging

## Abstract

**Background:** Digital subtraction angiography (DSA) remains the gold standard for assessing aneurysm morphology before and after treatment. While visual interpretation is common, quantitative image analysis remains underutilized in clinical practice. This study aimed to evaluate postoperative vascular changes in patients with middle cerebral artery (MCA) aneurysms using SymDIRECT-based pixel clustering on preoperative and postoperative DSA images. **Methods:** A total of 59 patients with unruptured MCA aneurysms were analyzed retrospectively. SymDIRECT clustering segmented angiographic images into four intensity clusters. Quantitative comparison of cluster pixel counts between pre- and postoperative images was performed. **Results:** Both neurosurgical clipping and endovascular treatment groups demonstrated significant reductions in medium- and high-intensity pixel clusters postoperatively, reflecting successful aneurysm occlusion. The background cluster increased post-treatment in most cases, with an average rise of over 14%, indicating effective anatomical exclusion of the aneurysm. **Conclusions:** SymDIRECT-based pixel clustering enables objective, pixel-level quantification of treatment response in DSA images. This approach may support standardized imaging follow-up protocols and improve reproducibility in neurovascular outcome assessment. Future integration with AI-based segmentation could facilitate real-time image interpretation.

## 1. Introduction

The successful treatment of brain aneurysms demands not only precise operative techniques but also robust methods of postoperative evaluation. Digital subtraction angiography (DSA) remains the gold standard for visualizing cerebral vasculature pre- and post-treatment due to its high spatial and temporal resolution and capacity to assess aneurysm occlusion and vascular remodeling [1]. Despite its strengths, visual interpretation of DSA remains largely qualitative, potentially limiting its ability to detect subtle yet clinically important changes in vascular architecture [2]. Post-treatment vascular remodeling is a dynamic process that can differ markedly between treatment modalities. While endovascular procedures may provoke hemodynamic shifts and gradual vessel wall adaptation, microsurgical clipping is generally considered to induce more localized and mechanically stable changes [3,4]. However, both require standardized, quantitative methods to monitor outcomes accurately. Clustering algorithms offer a promising solution by enabling segmentation of angiographic images based on pixel intensity, thereby providing a semi-automated and reproducible method of analyzing structural and hemodynamic changes. Recent studies have shown that fuzzy clustering and hybrid clustering techniques can achieve high precision in segmenting brain images and localizing vascular or tumorous lesions [5,6,7]. The SymDIRECT algorithm, a variant of the DIviding RECTangles method adapted for symmetric search spaces, facilitates pixel grouping into intensity-based clusters (background, low, medium, and high), allowing for detailed comparisons between pre- and postoperative images. This method differs from traditional supervised learning or convolutional neural network (CNN)-based segmentation by using interpretable pixel classification and deterministic clustering steps [8]. In the broader landscape of neuroimaging, machine learning, particularly clustering methods like fuzzy c-means, k-means, and transformer-based models, has proven effective for segmentation tasks in MRI and angiographic images, often outperforming manual segmentation and improving reproducibility [6,9,10]. These methods have enabled the extraction of clinically relevant features such as aneurysm volume, vessel wall thickness, and hemodynamic intensity changes over time. Notably, staged cluster transformer models for intracranial aneurysm segmentation from three-dimensional magnetic resonance angiography (3D MRA) have demonstrated high dice similarity coefficients, emphasizing the growing viability of cluster-based segmentation techniques in vascular imaging [6]. Furthermore, hybrid clustering approaches combining fuzzy logic, genetic algorithms, or entropy-based optimization show potential for robust performance even in noisy or low-contrast datasets [11,12].

This study applies SymDIRECT-based pixel clustering to pre- and postoperative DSA images of patients with middle cerebral artery (MCA) aneurysms treated via microsurgical clipping or endovascular techniques. By comparing pixel distribution across defined intensity clusters, we hypothesize that this method can reveal treatment-specific vascular remodeling patterns and potentially serve as an imaging biomarker of therapeutic efficacy [12,13,14].

## 2. Materials and Methods

This retrospective, single-center study analyzed DSA data from a consecutive series of 60 adult patients diagnosed with unruptured MCA aneurysms treated at the University Hospital Center Sestre Milosrdnice, Zagreb, Croatia, between January 2019 and June 2024. From an initial cohort of 103 patients, 43 were excluded based on predefined exclusion criteria. Patients were treated by either microsurgical clipping or endovascular embolization. All patients provided written informed consent following a comprehensive discussion of treatment options with a multidisciplinary team. Based on the intervention type, patients were divided into two cohorts. The clipping group included 30 patients (27 female and 3 male; mean age 57.5 ± 11.4 years) treated via microsurgical neck clipping. The endovascular group included 29 patients (24 female and 5 male; mean age 59.0 ± 9.0 years); one patient was excluded due to fatal thrombotic complications from non-compliance with dual antiplatelet therapy. Exclusion criteria included ruptured aneurysms, significant anatomical variation preventing proper visualization (e.g., vessel tortuosity or distal stenosis), lack of pre- and postoperative DSA, and deviation from prescribed therapy. All preoperative and follow-up DSA studies were performed using a Siemens Artis zee biplane system (Germany) with standardized settings across all scans. Preoperative DSA was acquired 1–3 days before intervention, while postoperative imaging was conducted 6–12 months after treatment. The average time interval between treatment and postoperative DSA imaging was 8.4 ± 1.7 months. In the endovascular group, the preoperative DSA was a separate diagnostic session, performed before the intervention for treatment planning. Intra-procedural images were excluded from the analysis due to variability in imaging parameters and the frequent presence of procedural artifacts. Image acquisition parameters, including contrast volume, frame rate, and field of view, were matched across timepoints to minimize technical variability. The same neurosurgeon and interventional neuroradiologist performed all procedures to maintain operator consistency. Each DSA study (anteroposterior and lateral projections) was exported in lossless 8-bit grayscale format (TIFF) and spatially cropped to isolate the MCA region. Cropping was performed manually using consistent anatomical landmarks (e.g., sphenoid ridge and MCA bifurcation) to maintain a fixed window size and spatial alignment across pre- and postoperative images. The projection angle and field of view were preserved to ensure comparable vessel coverage and minimize variability due to framing differences. Four representative static images per patient were selected and anonymized. These frames were selected from the arterial phase of each angiographic sequence based on maximal opacification of the MCA territory. Selection was performed by a neuroradiologist to ensure anatomical and contrast-phase consistency. Pre- and postoperative images were matched by projection view (AP and LAT), contrast timing, and vascular morphology to minimize temporal variability.

This study was conducted in accordance with the Declaration of Helsinki and approved by the Institutional Review Board of the University Hospital Center Sestre Milosrdnice, Zagreb, and the Faculty of Medicine, Josip Juraj Strossmayer University of Osijek, Croatia (No 602-04/21-08/07, approved on 14 January 2021). Written informed consent was obtained from all participants after a detailed explanation of the study protocol and treatment options.

### 2.1. Cluster Analysis

**Figure 1:**
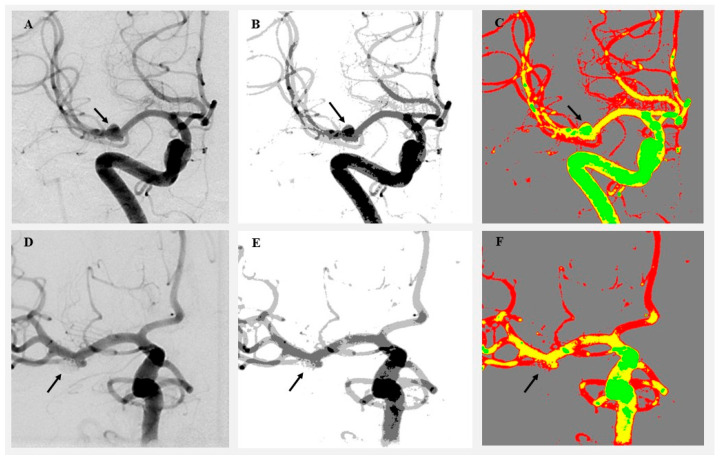
Visualization of angiographic image clustering in a patient treated by endovascular intervention. (**A**) Original preoperative DSA image showing a left MCA aneurysm (black arrow). (**B**) Preoperative grayscale cluster segmentation using the SymDIRECT algorithm. (**C**) Color-coded cluster image (gray = background, green = low, yellow = medium, and red = high intensity). (**D**) Original postoperative DSA image showing complete obliteration of the aneurysm. (**E**) Postoperative grayscale segmentation. (**F**) Postoperative color-coded cluster image demonstrating loss of high-intensity pixels at the aneurysm site. The black arrow indicates the location of the previously visualized aneurysm, now fully obliterated following endovascular coiling.

**Figure 2:**
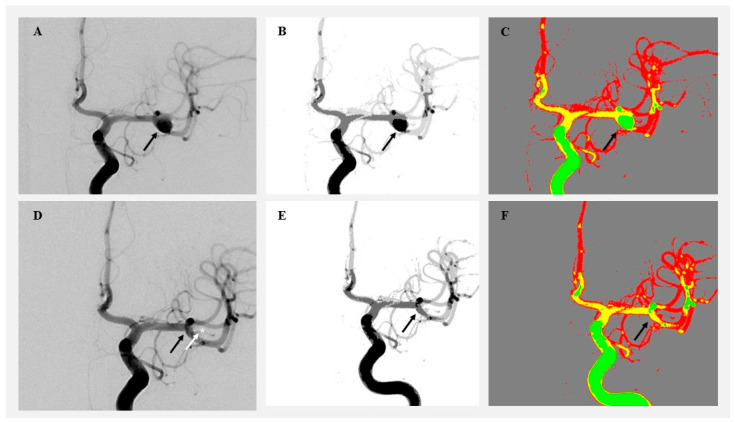
Visualization of angiographic image clustering in a patient treated by microsurgical clipping. (**A**) Original preoperative DSA image showing a right MCA aneurysm (black arrow). (**B**) Preoperative grayscale segmentation. (**C**) Preoperative color-coded cluster map highlighting aneurysm flow. (**D**) Postoperative DSA showing aneurysm occlusion. (**E**) Postoperative grayscale segmentation. (**F**) Postoperative color-coded image showing aneurysm obliteration (black arrow) and clip position (white arrow and white star).

Cluster-based image analysis was conducted using the SymDIRECT algorithm implemented in Wolfram Mathematica (version 14.1, Wolfram Research, Champaign, IL, USA). SymDIRECT is a symmetric variant of the DIviding RECTangles (DIRECTs) global optimization algorithm, designed to identify optimal pixel clusters in multidimensional spaces characterized by intensity regularity or symmetry [15,16]. Each angiographic image underwent preprocessing that included Gaussian and median filtering to suppress noise and enhance contrast. Intensity normalization was applied to equalize grayscale distribution. The algorithm then partitioned the grayscale range into symmetrical rectangles, seeking optimal intensity centers based on intra-cluster variance and spatial balance. These intensity centers were calculated automatically for each image; no manual thresholds or fixed values were applied. The final segmentation output included four clusters per image: background, low, medium, and high intensity. Pixels were assigned based on proximity to the nearest cluster centroid. Two visualization outputs were generated per image: a grayscale cluster map and a color-coded map (gray for background, green for low, yellow for medium, and red for high intensity). Canny edge detection was applied only to auxiliary visual overlays for quality control purposes and did not influence pixel classification. Any downsampling was limited to figure preparation and was not applied during cluster computation or quantitative analysis. These visualizations enabled clear identification of pre- and postoperative changes in aneurysm morphology and surrounding vasculature (Figure 1 and Figure 2).

The SymDIRECT algorithm used for clustering was implemented through Wolfram Mathematica’s built-in global optimization tools. This method is based on the DIRECT global optimization framework, originally developed for Lipschitzian functions [17] and later expanded for multidimensional and multiobjective search spaces [18]. Variants of DIRECT have also been successfully applied in image and signal processing contexts [19]. Although the core SymDIRECT routine is not open-source, it was adapted to this application using a custom pipeline comprising Gaussian and median filtering, grayscale normalization, objective function tuning, and pixel-wise intensity classification. Cluster boundaries were determined by minimizing intra-cluster variance across four symmetric intensity groups. Pixels were assigned to one of four clusters (background, low, medium, and high) and color-coded accordingly (gray, green, yellow, and red). The full pipeline, including data preparation and segmentation logic, is described in Supplementary File S1.

To refine boundary detection and prevent pixel misclassification, the Canny edge detection algorithm was applied in selected cases [20]. This method has been widely validated for structural edge enhancement in neuroimaging. Downsampling was used for high-resolution images to optimize memory usage and algorithm speed. The SymDIRECT clustering pipeline remains manual and labor-intensive. On average, each image required approximately 30–45 min to preprocess, segment, and validate, necessitating a trained analyst familiar with both neurovascular anatomy and clustering algorithms.

All clustered images were independently assessed by two neurovascular imaging experts, each with substantial experience in DSA interpretation. Each reviewer evaluated the segmentation accuracy based on visual alignment with known vascular anatomy and aneurysm regions. In cases of disagreement, consensus was reached through joint image review and discussion. Inter-rater agreement was quantified using Cohen’s kappa coefficient, yielding κ > 0.85, confirming high reproducibility and reliability of the segmentation process.

To assess the robustness and reproducibility of the clustering pipeline, an inter-observer consistency test and sensitivity analysis were conducted on a subset of 10 patients. These tests confirmed that minor variations in frame selection and ROI definition did not significantly affect pixel distribution or statistical outcomes. Details are provided in Supplementary File S2.

### 2.2. Statistical Analysis

All statistical analyses were performed using MedCalc Statistical Software (version 12.5.0, Ostend, Belgium). Continuous variables were expressed as mean ± standard deviation for normally distributed data or as median and interquartile range (IQR) for non-normally distributed data. Normality was assessed using the Shapiro–Wilk test. To compare preoperative and postoperative values within each treatment group, the Wilcoxon Signed-Rank test was used for paired non-parametric data. Between-group comparisons (microsurgical vs. endovascular) were performed using the Student’s *t*-test or the Mann–Whitney U test, depending on data distribution. A *p*-value < 0.05 was considered statistically significant.

## 3. Results

Postoperative aneurysm obliteration was confirmed in all patients included in the final analysis, based on follow-up DSA imaging performed 6 to 12 months after treatment. In both the microsurgical clipping group and the endovascular group, 100% of patients demonstrated complete aneurysm obliteration without residual neck or sac filling. Cluster-based analysis of DSA images revealed distinct and quantifiable differences in grayscale pixel distributions before and after aneurysm treatment. By segmenting each angiographic image into four defined pixel intensity clusters using the SymDIRECT algorithm, we objectively assessed morphological and flow-related changes in cerebral vasculature, offering a novel, data-driven lens for evaluating treatment response.

### 3.1. Pixel-Level Remodeling Following Endovascular and Neurosurgical Treatment

In the endovascular group, a consistent postoperative increase in Cluster 1 pixels (background) was observed, trending toward statistical significance (*p* = 0.056). This increase averaged +11.8% and likely represents angiographic “silencing” of previously perfused aneurysmal regions that no longer fill with contrast and are thus recategorized as background by the segmentation algorithm. Additionally, Cluster 3 (medium intensity) showed a postoperative increase (*p* = 0.08), suggesting subtle redistribution of contrast flow in the surrounding vascular network. Conversely, Cluster 4 (high intensity) decreased by −21.4% (*p* = 0.18), potentially indicating reduced focal turbulence or pooling, a common feature of pretreated aneurysms.

The microsurgical clipping group exhibited relatively stable pixel distributions between pre- and postoperative images. No statistically significant changes were observed, although Cluster 4 pixels demonstrated a mild increase of +19.7% (*p* = 0.11), possibly due to residual contrast signal at the clip site or metallic artifact interference with segmentation. Cluster 1 showed a +14.2% increase (*p* = 0.23), suggesting partial occlusion-related signal dropout. These results imply that endovascular interventions may induce more dynamic postoperative grayscale redistribution compared to surgical clipping, which produces relatively stable angiographic profiles.

When comparing postoperative images between groups, Cluster 1 pixels were significantly more numerous in the endovascular group (*p* < 0.01), consistent with more pronounced obliteration of the aneurysmal sac and reduced contrast enhancement. Conversely, Cluster 4 was significantly more prominent in the surgical group (*p* = 0.04), likely reflecting either metallic artifact from clips or high-density signal near the clip site (Table 1).

### 3.2. Clinical Interpretation of Pixel Redistribution

**Figure 3:**
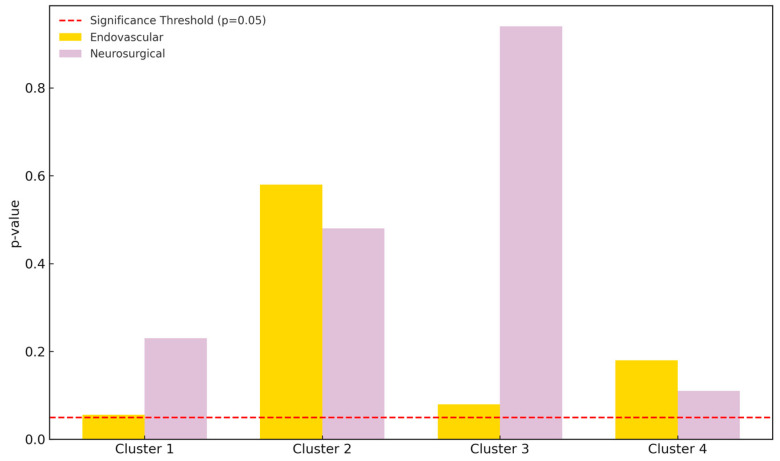
Pixel count comparison between preoperative and postoperative DSA images using SymDIRECT clustering. Cluster 1 represents background, while clusters 2, 3, and 4 represent low-, medium-, and high-intensity vascular structures, respectively. The endovascular treatment group is shown in yellow; the neurosurgical group in light purple. A dashed red line marks the threshold of statistical significance (*p* = 0.05).

Cluster analysis revealed consistent and measurable pixel distribution changes between pre- and postoperative DSA images in both treatment groups. The most pronounced change occurred in Cluster 1 (background), with increases of +14.2% in the surgical group and +11.8% in the endovascular group (Figure 3). This suggests a substantial reduction in perfused vascular structures, indicating successful anatomical exclusion of the aneurysmal sac. Clusters 3 and 4 (medium and high intensity) showed overall decreases. Cluster 4, associated with high-intensity flow signals and turbulent regions, decreased by −21.4% in the endovascular group and −19.7% in the surgical group. These reductions likely correspond to the loss of pulsatile inflow or contrast pooling post-treatment. Changes in Cluster 2 (low intensity) were minor in both groups (−4.9% endovascular; −6.3% surgical), reflecting generalized signal homogenization rather than focal vascular change (Table 1). These observations reinforce the potential of pixel-based segmentation to capture treatment-induced hemodynamic remodeling. Increases in background pixel counts reflect flow silencing and anatomical occlusion of the aneurysm, while reductions in higher-intensity clusters suggest decreased intraluminal turbulence or redirected perfusion.

## 4. Discussion

Cluster analysis assumes the grouping of a set of objects in such a way that the objects in the same group (cluster) are more similar (defined and determined by the analyst) to each other than to those in other groups (clusters). Cluster analysis is widely used across scientific disciplines, including pattern recognition [21], image analysis [22,23], bioinformatics [24,25], and data science applications like compression and retrieval [26,27].

In medicine, cluster analysis is commonly employed in biostatistical research to define patient phenotypes and predict clinical outcomes based on anamnestic or phenotypic characteristics [28,29,30]. In imaging, cluster methods are used to classify and interpret MRI, CT, and radiographic data by grouping similar structural features to derive clinically meaningful categories [31,32,33]. However, current hemodynamic studies often rely on computational models or non-invasive measurement techniques such as ultrasound [34,35]. Few clinical studies incorporate cluster analysis into the direct evaluation of vascular imaging data. A comprehensive search of the literature found no existing studies applying cluster analysis to raw DSA imaging for hemodynamic interpretation. Our study is, therefore, novel in using SymDIRECT-based clustering to assess treatment-induced vascular changes directly from DSA images. Our findings indicate that pixel cluster changes, especially in clusters 3 and 4, which reflect medium- and high-intensity regions, are indicative of hemodynamic remodeling following intervention. This is consistent with known principles of hemodynamic redistribution after aneurysm obliteration [36,37]. Cluster 4, in particular, may represent zones of higher blood flow or residual luminal signal, and its reduction suggests effective occlusion. Cluster 2, representing lower intensity regions, showed subtle but consistent shifts, indicating alterations in adjacent flow territories. These findings, while preliminary, support the idea that grayscale clustering can reflect nuanced vascular dynamics. The observed inconsistencies in cluster trends across patients likely reflect variations in vascular anatomy, aneurysm morphology, and flow dynamics. Postoperative aneurysm obliteration was confirmed in all patients included in the final analysis, based on follow-up DSA imaging performed 6 to 12 months after treatment. Both the microsurgical clipping and endovascular groups demonstrated 100% complete aneurysm obliteration without residual neck or sac filling. This exceptionally high obliteration rate reflects careful patient selection and the effective application of current treatment techniques. While long-term durability remains an important consideration, our findings align with other short-term outcome studies in MCA aneurysms. For example, the prospective study by Khamis et al. [38] reported early complete occlusion in both clipping and coiling groups. Similarly, the meta-analysis demonstrated comparable early obliteration success with both techniques [39]. Longer-term angiographic surveillance would, however, be necessary to confirm the sustained stability of these outcomes. These inter-individual differences further support the use of cluster-based metrics as patient-specific, image-derived indicators of vascular response. This aligns with the broader trend toward personalized medicine, where individualized image-based biomarkers could inform follow-up strategy and treatment choice. A strength of the method lies in its precision: the clustering was consistent across timepoints and imaging sets due to standardized acquisition parameters. Additionally, inter-rater reliability was high, supporting reproducibility. Compared to visual DSA analysis, our method provides quantitative metrics that are less subject to observer variability. While MRI and CT angiography are increasingly utilized in follow-up imaging, DSA remains the modality of choice in this study due to its consistently high spatial and temporal resolution and its established clinical use in aneurysm evaluation. We acknowledge, however, that recent advancements such as photon-counting CT may narrow this gap and deserve further comparative exploration. Another novelty is the application of SymDIRECT, a global optimization algorithm particularly suited to symmetric intensity distributions [40]. It outperformed heuristic or fuzzy clustering approaches in consistency and convergence rate. Compared to fuzzy clustering, which may suffer from ambiguity in boundary definition [21], SymDIRECT ensures robust segmentation even in complex angiographic images, as shown in our cohort. However, SymDIRECT-based segmentation remains time-intensive and is not yet suitable for routine clinical use. Each image requires manual preprocessing, segmentation, and interpretation, often taking 30–45 min per case. Without a standardized or automated protocol, widespread implementation in clinical radiology remains a challenge. This reinforces the need for interdisciplinary collaboration and future integration with machine learning pipelines [41]. With further development, clustering could be incorporated into a fully automated pipeline by integrating convolutional neural networks such as the U-Net architecture, which has shown strong performance in medical image segmentation [42]. In this framework, the U-Net could first generate vessel segmentation masks, followed by automated pixel intensity clustering, enabling real-time quantification of vascular remodeling across standardized DSA inputs.

To facilitate clinical adoption, future study should aim to develop an automated analysis pipeline. A potential framework could include deep learning-based preprocessing, automated grayscale normalization, and clustering through convolutional architectures. Edge-detection techniques such as Canny filters [20] could further refine cluster boundaries, improving segmentation accuracy in vascular regions with complex topology. Moreover, increasing the number of clusters may yield higher resolution and more granular mapping of vascular territories. Integrating morphological descriptors like vessel diameter, curvature, or tortuosity may enable fusion of structural and hemodynamic features, enriching clinical interpretation. Finally, extending the clustering to dynamic DSA sequences, rather than single frames, would allow modeling of temporal flow parameters such as contrast arrival time or pulsatility, advancing toward a true functional imaging tool. Overall, SymDIRECT-based clustering of DSA images represents a promising method for evaluating vascular changes after aneurysm treatment. Though not yet optimized for clinical workflow, it holds strong potential for enhancing the precision and objectivity of neurovascular imaging assessment.

This study has several limitations that should be acknowledged. First, the relatively small sample size limits the statistical power and generalizability of our findings. The subgroup size after stratification (approximately 30 patients per treatment group) further reduces the power of between-group comparisons and limits the interpretation of statistical trends. Although cluster intensity differences were consistently observed, especially in background and high-intensity regions, statistical significance was not reached in all comparisons, likely due to sample size constraints and inter-individual anatomical variability. Second, the analysis was based exclusively on static 2D DSA frames. No temporal information from the dynamic DSA series (e.g., contrast arrival time and flow rate) was included, which limits the method’s sensitivity to detect real-time hemodynamic phenomena. Inclusion of three-dimensional DSA reconstructions or dynamic perfusion sequences would further enhance the ability to capture temporal and spatial aspects of flow, offering a more comprehensive understanding of hemodynamic alterations following treatment. Additionally, the SymDIRECT algorithm was not applied to immediate post-intervention DSA images. Including such early imaging could allow assessment of acute procedural effects, and future studies should explore its use for intra-procedural evaluation of treatment success. Third, the SymDIRECT algorithm assumes relatively symmetrical intensity distributions and was applied only to grayscale pixel values. The approach does not account for spatial vascular morphology, such as vessel diameter, curvature, or aneurysm shape, factors that are clinically relevant for risk stratification and treatment planning. Integrating these morphological descriptors into clustering logic could enhance diagnostic accuracy and anatomical relevance. Fourth, while preprocessing steps (e.g., Gaussian filtering) were employed to reduce artifacts and normalize intensities, image quality remained a critical determinant of segmentation success. The algorithm’s performance may vary across scanners, acquisition protocols, and contrast timing, potentially limiting generalizability without standardized input preparation. Fifth, there is no existing automated or clinical-grade pipeline for implementing SymDIRECT-based clustering in a real-world hospital environment. Manual preprocessing and segmentation are labor-intensive, require domain-specific expertise, and are not scalable for large datasets or routine workflows. Despite high inter-rater agreement, region-of-interest selection and thresholding introduce inherent subjectivity. Finally, aneurysm morphology (e.g., dome size and neck width) was not explicitly measured or included in the clustering analysis. These variables likely influence hemodynamic patterns and could interact with intensity-based segmentation in ways not accounted for in the current model. Future research should therefore prioritize the development of automated, artificial intelligence (AI)-powered clustering pipelines, integration of morphometric and temporal data, and validation across larger, heterogeneous patient cohorts.

## 5. Conclusions

This study demonstrates that SymDIRECT-based cluster analysis of DSA images is a feasible, reproducible, and innovative approach for evaluating cerebral hemodynamics following MCA aneurysm treatment. By quantifying changes in grayscale pixel distributions before and after intervention, this method offers a structured and objective lens to assess the vascular impact of both endovascular and microsurgical approaches. The strength of this approach lies in its application of a global optimization algorithm to angiographic data, allowing the detection of subtle grayscale redistribution patterns linked to blood flow reorganization, thrombus formation, or device-related signal changes. The method was particularly effective in identifying postoperative changes in background and high-intensity pixel clusters, aligning with known biological effects of aneurysm occlusion. Although current barriers, such as lack of automation, limited 2D scope, and absence of morphometric integration, hinder widespread clinical implementation, these challenges are surmountable. Advancements in artificial intelligence, computer vision, and clinical imaging standardization offer a clear path forward.

This study lays the foundation for the integration of AI-driven pixel clustering into clinical DSA analysis workflows, potentially enabling real-time, quantitative vascular assessment following aneurysm treatment and ushering in a new era of personalized neurovascular imaging.

## Figures and Tables

**Table 1:** Combined descriptive statistics of pixel clusters pre- and postoperatively in both groups and statistical significance of cluster changes within and between groups.

Cluster	Endo Pre (Mean ± SD)	Endo Post (Mean ± SD)	*p* (Endo Pre→Post)	Neuro Pre (Mean ± SD)	Neuro Post (Mean ± SD)	*p* (Neuro Pre→Post)	*p* (Post Endo vs. Neuro)
Cluster 1	5212 ± 480	5778 ± 510	0.056	4986 ± 462	5010 ± 447	0.23	<0.01
Cluster 2	1514 ± 278	1492 ± 261	0.58	1486 ± 250	1478 ± 244	0.48	NS
Cluster 3	624 ± 110	713 ± 130	0.08	658 ± 128	649 ± 120	0.94	NS
Cluster 4	136 ± 45	119 ± 42	0.18	142 ± 41	158 ± 39	0.11	0.04

Legend: Cluster 1 = background; Cluster 2 = low-intensity; Cluster 3 = medium-intensity; Cluster 4 = high-intensity pixel areas. Endo Pre = preoperative endovascular group; Endo Post = postoperative endovascular group; Neuro Pre = preoperative neurosurgical group; Neuro Post = postoperative neurosurgical group.

## Data Availability

All data generated or analyzed during this study are included in this published article. All tables and figures were created by the authors and are original.

## Supplementary Materials

The following supporting information can be downloaded at: https://www.mdpi.com/article/10.3390/diagnostics15162036/s1, Supplementary File S1. Code for SymDIRECT-Based Pixel Clustering Pipeline; Supplementary File S2. Inter-observer and Sensitivity Analysis of Clustering Robustness.

## References

[1] Rhoton A.L. (2007). The supratentorial arteries. Neurosurgery.

[2] Raymond J., Guilbert F., Weill A., Georganos S.A., Juravsky L., Lambert A., Lamoureux J., Chagnon M., Roy D. (2003). Long-term angiographic recurrences after selective endovascular treatment of aneurysms. Stroke.

[3] Wiebers D.O. (2003). Unruptured intracranial aneurysms: Natural history, clinical outcome, and risks of surgical and endovascular treatment. Lancet.

[4] Fiorella D., Albuquerque F.C., Han P., McDougall C.G. (2004). Preliminary experience using the Neuroform stent for the treatment of cerebral aneurysms. Neurosurgery.

[5] Shishegar R., Joshi A., Tolcos M.D., Walker W., Johnston L. Clustering Algorithm for Brain Image Segmentation. Proceedings of the International Conference on Neural Information Processing.

[6] Guo L., Liang Y., Guo R., Cao Z., Ye J., Lai X. (2024). Staged cluster transformers for intracranial aneurysms segmentation from 3D MRA. Int. J. Imaging Syst. Technol..

[7] Hooda H., Verma O. (2022). Fuzzy clustering using gravitational search algorithm for MRI brain segmentation. Multimed. Tools Appl..

[8] Gokcay E., Príncipe J. A new clustering algorithm for segmentation of magnetic resonance images. Proceedings of the IEEE EMBS Conference.

[9] Shasidhar M., Raja V.S., Kumar B.V. MRI Brain Image Segmentation Using Modified Fuzzy C-Means. Proceedings of the IEEE Conference on Communication Systems and Network Technologies (CSNT).

[10] Yang Y. Effective fuzzy clustering algorithm for brain MR image segmentation. Proceedings of the World Congress on Intelligent Control and Automation (WCICA).

[11] De Oliveira G., Varoto R., Cliquet A. Brain tumor segmentation in MRI using genetic algorithm clustering and AdaBoost Classifier. Proceedings of the IEEE International Conference on Systems, Man, and Cybernetics.

[12] Molyneux A., Kerr R., Stratton I., Sandercock P., Clarke M., Shrimpton J., Holman R. (2002). International subarachnoid aneurysm trial (ISAT). Lancet.

[13] Cebral J.R., Ollikainen E., Chung B.J., Mut F., Sippola V., Jahromi B.R., Tulamo R., Hernesniemi J., Niemelä M., Robertson A. (2017). Flow Conditions in the Intracranial Aneurysm Lumen Are Associated with Inflammation and Degenerative Changes of the Aneurysm Wall. AJNR Am. J. Neuroradiol..

[14] Wang J., Wang N., Wang R. Research on medical image processing based on ITK algorithm. Proceedings of the 3rd International Conference on Mechatronics and Industrial Informatics.

[15] Jones D., Martins J. (2020). The DIRECT algorithm: 25 years later. J. Glob. Optim..

[16] Shah K., Fu H., Kosorok M. (2021). Stabilized direct learning for efficient estimation of individualized treatment rules. Biometrics.

[17] Jones D.R., Perttunen C.D., Stuckman B.E. (1993). Lipschitzian optimization without the Lipschitz constant. J. Optim. Theory Appl..

[18] Custódio A.L., Madeira J.F.A., Rosa S., Vicente L.N. (2010). Direct multisearch for multiobjective optimization. SIAM J. Optim..

[19] Lera D., Sergeyev Y.D. (2016). Acceleration of univariate global optimization algorithms working with Lipschitz functions and Lipschitz gradients. SIAM J. Optim..

[20] Canny J. (1986). A computational approach to edge detection. IEEE Trans. Pattern Anal. Mach. Intell..

[21] Baraldi P., Blonda P. (1999). A survey of fuzzy clustering algorithms for pattern recognition. IEEE Trans. Syst. Man Cybern. B.

[22] Sathya B., Manavalan R. (2011). Image Segmentation by Clustering Methods: Performance Analysis. Int. J. Comput. Appl..

[23] Singh K.K., Singh A. (2010). A Study of Image Segmentation Algorithms for Different Types of Images. Int. J. Comput. Sci. Issues.

[24] Wismüller A., Lange O., Dersch D.R., Leinsinger G.L., Hahn K., Pütz B., Auer D. (2002). Cluster Analysis of Biomedical Image Time-Series. Int. J. Comput. Vis..

[25] Wong K.-P., Feng D., Meikle S.R., Fulham M.J. (2002). Segmentation of dynamic PET images using cluster analysis. IEEE Trans. Nucl. Sci..

[26] Ng R.T., Han J. Efficient and Effective Clustering Methods for Spatial Data Mining. Proceedings of the 20th International Conference on Very Large Data Bases (VLDB).

[27] Ezugwu A.E., Ikotun A.M., Oyelade O.O., Abualigah L., Agushaka J.O., Eke C.I., Akinyelu A.A. (2022). A comprehensive survey of clustering algorithms: State-of-the-art machine learning applications. Eng. Appl. Artif. Intell..

[28] Sharma A., Zheng Y., Ezekowitz J.A., Westerhout C.M., Udell J.A., Goodman S.G., Armstrong P.W., Buse J.B., Green J.B., Josse R.G. (2022). Cluster Analysis of Cardiovascular Phenotypes in Patients With Type 2 Diabetes. Diabetes Care.

[29] Weatherall M., Shirtcliffe P., Travers J., Beasley R. (2010). Use of cluster analysis to define COPD phenotypes. Eur. Respir. J..

[30] Gilbody S., Bower P., Torgerson D., Richards D. (2008). Cluster randomized trials and care for depression. J. Clin. Epidemiol..

[31] Eddy R.L., McIntosh M.J., Matheson A.M., McCormack D.G., Licskai C., Parraga G. (2022). Pulmonary MRI and Cluster Analysis Help Identify Novel Asthma Phenotypes. J. Magn. Reson. Imaging.

[32] Borri M., Schmidt M., Powell C., Koh D.M., Riddell A., Partridge M., Bhide S., Nutting C., Harrington K., Newbold K. (2015). Characterizing Heterogeneity within Head and Neck Lesions Using Cluster Analysis of Multi-Parametric MRI Data. PLoS ONE.

[33] Papiris S., Georgakopoulos A., Papaioannou A., Pianou N., Kallergi M., Kelekis N., Gialafos H., Manali E., Chatziioannou S. (2020). Emerging phenotypes of sarcoidosis based on 18F-FDG PET/CT: A hierarchical cluster analysis. Expert Rev. Respir. Med..

[34] Fraser A.G., Claus P. (2017). Beyond Bernoulli: Estimation of Aortic Stenosis Severity. Circ. Cardiovasc. Imaging.

[35] Galarce F., Lombardi D., Mula O. (2021). Reconstructing haemodynamics from Doppler ultrasound imaging. Int. J. Numer. Methods Biomed. Eng..

[36] Anderson D., Tannehill J.C., Pletcher R.H. (2020). Computational Fluid Mechanics and Heat Transfer.

[37] Zhang Y., Fan J., Xiu Y., Zhang L., Chen G., Fan J., Lin X., Ding C., Feng M., Wang R. (2022). Numerical simulation flow dynamics of an intracranial aneurysm. Biomed. Mater. Eng..

[38] Khamis M., Ibrahim H., Elsayed A.M., Tawadros S.R., Mohamed M.H.A., El-Bahy K. (2025). Comparative analysis of microsurgical clipping versus endovascular coiling for middle cerebral artery aneurysms: A prospective study at Ain Shams University hospitals. Egypt. J. Neurosurg..

[39] Alreshidi M., Cote D.J., Dasenbrock H.H., Acosta M., Can A., Doucette J., Simjian T., Hulou M.M., Wheeler L.A., Huang K. (2018). Coiling Versus Microsurgical Clipping in the Treatment of Unruptured Middle Cerebral Artery Aneurysms: A Meta-Analysis. Neurosurgery.

[40] De Beukelaer F., Wuyts L., De Beukelaer S., Van Hedent S., Nikoubashman O., Wiesmann M., Veldeman M., Pjontek R., Höllig A., Ridwan H. (2025). Photon-counting CT-angiography in comparison to digital subtraction angiography for assessing intracranial aneurysms after coiling or clipping. Neuroradiology.

[41] Choi K.S., Sunwoo L. (2022). Artificial Intelligence in Neuroimaging: Clinical Applications. Investig. Magn. Reson. Imaging.

[42] Ronneberger O., Fischer P., Brox T. (2015). U-Net: Convolutional Networks for Biomedical Image Segmentation. Medical Image Computing and Computer-Assisted Intervention—MICCAI 2015.

