# Supplementary material for: Quantitative DSA Analysis of MCA Aneurysms Using SymDIRECT Pixel Clustering: A Novel Framework for Objective Post-Treatment Evaluation
Source: Diagnostics (Basel). 2025 Aug 14;15(16):2036. doi: 10.3390/diagnostics15162036 (PMC12385858; doi:10.3390/diagnostics15162036)
Supplement: Supplementary file 1 [file diagnostics-15-02036-s001.zip › diagnostics-3723725-supplementary.pdf]

Supplementary File S1. Code for SymDIRECT-Based Pixel Clustering Pipeline.

This code was prepared to enhance reproducibility and transparency of the SymDIRECT-based clustering methodology used in this study.

Input: Static 8-bit grayscale DSA images in TIFF format (2 projections per patient: AP and lateral)
Output: Grayscale cluster maps, Color-coded cluster maps, Pixel count per cluster (CSV format) for statistical analysis
Steps:
1. Image Acquisition & Preparation
For each patient, load preoperative and postoperative DSA images (AP and LAT views); crop the image to isolate the middle cerebral artery (MCA) region of interest.
2. Preprocessing
Apply Gaussian filter (σ = 1.5) to smooth local intensity variations; Apply median filter (kernel size = 3x3) to reduce salt-and-pepper noise; Perform histogram normalization to equalize contrast distribution across images; Optionally downsample image (factor 0.5–0.75) to improve computational efficiency.
3. Clustering Algorithm (SymDIRECT)
Define objective function based on intra-cluster intensity variance minimization; Initialize 4 symmetrical intensity cluster centers within the grayscale range [0–255]; Use Wolfram Mathematica's SymDIRECT optimizer to iteratively adjust cluster centers; Terminate clustering when Intra-cluster variance change < ε (e.g., 1e-3); Maximum number of iterations reached (e.g., 1000)
4. Pixel Classification
For each pixel in the image, assign the pixel to the nearest cluster center based on absolute intensity distance; store cluster ID (1 = background, 2 = low, 3 = medium, 4 = high)
5. Edge Enhancement (Optional)
In selected cases (e.g., unclear boundaries), Canny edge detection is applied to refine vessel outlines.
6. Output Generation
Create grayscale cluster map (each pixel labeled by cluster ID); create color-coded map using fixed scheme: Cluster 1: gray (background), Cluster 2: green (low intensity), Cluster 3: yellow (medium intensity), Cluster 4: red (high intensity); export both images as high-resolution PNG files.
7. Data Extraction
Count the number of pixels in each cluster per image; export pixel counts to a structured table (CSV format), organized by patient ID and timepoint (pre/post).
8. Validation
Independent visual review by two observers, in case of disagreement, perform a joint consensus review; calculate inter-rater agreement using Cohen's kappa (target κ > 0.85)

Notes:
The SymDIRECT optimizer used is part of Wolfram Mathematica's global optimization package.
Full automation was not available; segmentation required manual parameter tuning and verification.
All code was executed on a local workstation (Intel i7 CPU, 32 GB RAM, NVIDIA RTX GPU, Mathematica 14.1).
Edge detection and resolution reduction were only used for figure generation and visual quality control.

Supplementary File S2. Inter-observer and Sensitivity Analysis of Clustering Robustness.

1. Inter-observer consistency test

Sample: 10 patients (5 microsurgical, 5 endovascular)

Observers: Primary author vs. independent neuroradiologist

Process:

- Key frame selection
- ROI delineation
- SymDIRECT pixel clustering

Results:

Cluster	Mean % difference	p-value
Cluster 1	3.2% ± 1.1	0.21
Cluster 2	2.4% ± 0.9	0.36
Cluster 3	4.1% ± 1.4	0.19
Cluster 4	3.8% ± 1.3	0.27

2. Sensitivity analysis

Process: ROI boundaries shifted ±5% (up, down, left, right)

Effect on clustering results:

Maximum deviation: 3.2%

Statistical outcomes remained unchanged

Conclusion

These tests confirm that the method is robust to minor inter-operator and ROI definition variability. Clustering outcomes are consistent, supporting the reproducibility of the proposed imaging pipeline.
